# Origin of Evolution *versus* Origin of Life: A Shift of Paradigm

**DOI:** 10.3390/ijms12063445

**Published:** 2011-06-01

**Authors:** Marc Tessera

**Affiliations:** 2 Avenue du 11 Novembre 1918, Meudon 92190, France; E-Mail: marc.tessera@wanadoo.fr; Tel.: +33-6-86-46-60-94

**Keywords:** origin of life, origin of evolution, open far-from-equilibrium systems, lipidic vesicles, heredity, hydrothermal vents

## Abstract

The question of the primordial ancestor must be approached through the search for the origin of evolution, not through the search for the origin of life. There is a major issue with the concept of life because it is impossible to define, thus is not a scientific but a metaphysical concept. On the contrary, evolution may be defined by as few as three conditions. These do not necessarily involve biopolymers. However, such an approach must give clues to explain the emergence of distinct lineages to allow Darwinian natural selection. A plausible solution exists within an autotrophic lipidic vesicle-based model that is presented. The model requires the existence of hydrothermal sites such as the Lost City Hydrothermal Field leading to specific constraints. For this reason Mars and Europa may be questioned as possible cradles of evolution. If we replace the search for the origin of life by the one for the origin of evolution our priority first is to find a consensus on the minimal conditions that would allow evolution to emerge and persist anywhere in the universe.

## 1. Introduction

A consensus among the specialists (chemists, geochemists, biochemists, biologists, exo/astrobiologists, computer scientists, philosophers and historians of science) in the search for the origin of life seems to be that there is an “obvious need for a definition of life”. However the same specialists admit that “there are hundreds of working, conventional definitions of life within scientific discourse, but none has been able to achieve a consensus [1]. During a recent conference held in Paris in 2008 entitled “Defining Life”, it was acknowledged that “contemporary scientists remain exceedingly dependent upon the spontaneous intuition of life” [2]. The concept of life is “too vague and general, and loaded with a number of historical, traditional, religious values” [3]. Although life is “a useful word in practice”, it is “not a scientific concept” [2]. Actually the concept of life is related to an indefinable state. Any definition of life is subjective and arbitrary: the boundary between living and non-living systems or pinpointing the moment when non living systems would have become living. For instance, saying that virus or prions or lipidic vesicles with the capacity of evolving are living systems (or not) adds nothing more than the definition of life one would propose. Therefore, as the distinction between living and non living systems is a matter of belief and not science, it is hopeless to study this indefinable state in relation to a metaphysical question. Complex systems that are unable to evolve have fixed dynamic structures (e.g., cyclonic/anticyclonic systems). In contrast, complex systems that are capable of evolving have the chance of undertaking a long history of changes. Most of the so-called living systems appear markedly different from inanimate systems because they are the end-products of a continuum of changes spanning billions of years.

## 2. The Primordial Ancestor and the Conditions for Evolution

### 2.1. What Is the Question?

But what is the question, actually? Most specialists of the origin of life agree that all extant and past terrestrial systems that have stemmed from closely related parent systems have a primordial ancestor. It is therefore crucial to elucidate the process by which lineages began and continued, and that process is, obviously, evolution. The extant and past terrestrial systems that have evolved since the primordial ancestor are self-organizing systems. Although the concept of self-organization is not easy to define [4] it can be described as the spontaneous emergence of open non-equilibrium structural organization on a macroscopic level, due to the collective interactions between a large number of (usually simple) microscopic subsystems [5]. In addition, this open non-equilibrium structural organization has to maintain its organization: as pointed out by Schrödinger, reconciling self-organization with thermodynamics, maintaining a local level of organization is only possible in the context of a non-equilibrium setting [4,6]. Experimentally speaking, open far from equilibrium systems can be understood through the notion of deterministic chaos systems, specifically systems that involve Belousov-Zhabotinsky-like chemical reactions. These systems are based on mutually catalytic chemical reactions and maintain their far from equilibrium state if they are provided with a continuous supply of the needed ingredients and drained from their waste products [7]. On the theoretical part, Prigogine made a major contribution to this approach, particularly regarding the potential of such systems to increase their level of organization (*i.e.*, evolution of the systems). He has advanced a morphogenetic perspective compatible with an internalist view point, wherein microscopic dynamics that place in open, far from equilibrium systems is considered to act as a source of increasing organization [8].

### 2.2. Three Conditions for Evolution

Within the paradigm of open, far from equilibrium systems that should maintain their level of organization, it is possible to only envisage three conditions that would permit the systems to evolve: (1) Local conditions that allow the emergence of open non-equilibrium structural systems, organized on a macroscopic level, generated by a flow of matter and energy that is continuously supplied. These open far-from-equilibrium systems can maintain themselves far-from-equilibrium because they are able to use the matter and energy supplied by the favourable local environment; (2) The systems must be able to self-replicate; (3) The systems must be capable of acquiring heritable structure/function properties that are relatively independent from the local environment, *i.e.*, the fact that they belong to a specific lineage should not depend on the nature of the nutriments they receive from the local environment [9]. This last condition is required for the emergence of distinct lineages allowing Darwinian natural selection. I do not mention a possible fourth condition that I mentioned in a previous article and that corresponds to a form of mutation: “These properties may change sporadically while remaining transmissible to the descendants” [9]. Actually this fourth condition is not required to allow room for selection if the potential of the systems is very large for the emergence of new distinct lineages. One interesting feature of this set of three conditions is that it does not necessarily involve a genetic component related to nucleic acids.

### 2.3. Two Schools of Thought

There are presently two schools of thought regarding the above matter. Some specialists are reluctant to adhere to the concept of systems lacking nucleic acids. They consider that only nucleic acid-related genetic components can display properties reflecting the role that Darwinian natural selection and, in general, evolutionary processes, have played in shaping the characteristics of the so-called living systems [10]. However, epigenetic mechanisms based on structural inheritance systems exist in present organisms [11]. Moreover the problem of the synthesis of genetic polymers within the constraints of prebiotic chemistry presently remains “a major unanswered issue” [10]. Actually, even if the abiotic synthesis of genetic polymers was possible, the question would not be solved as the issue is not to synthesize specific molecules. It is the need of the spontaneous emergence of non-equilibrium self-organizing systems with evolvable capacity. Other scientists prefer therefore to support the idea that these non-equilibrium self-organizing systems have dynamic properties that exist in a state close to chaotic behaviour allowing the emergence of autocatalytic cycles prior to the appearance of nucleic acids and genetic systems [10]. Within this approach, a model based on heterogeneous assemblies of diverse lipid-like mutally catalytic amphiphilic molecules has been conceptualized by Doron Lancet at Weizmann in Israel; the GARD system simulations. According to the authors, the model would have some capacity for evolution [12]. However, it has recently been demonstrated that replication of compositional information such as the one described by Segré and Lancet is so inaccurate that fitter compositional genomes cannot be maintained by selection and the system therefore lacks evolvability [13]. The approach of open non-equilibrium self-organizing systems without resorting to genetic polymers is confronted with the problem of heredity, *i.e.*, the need for the systems to acquire heritable structure/function properties (condition 3). For example, in the lipidic vesicle model, experimental data shows that the vesicles can grow and bud (a kind of reproduction) by exploiting nutrient and energy gradients while producing entropy even before a directed synthetic system is established [14–17]. However these vesicles cannot evolve, as they do not fulfil condition 3.

## 3. A Plausible Solution within a Lipidic Vesicle-Based Model

### 3.1. Proposal Model

**Figure 1:**
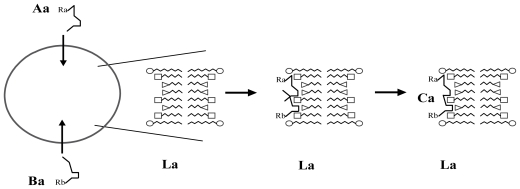
Schematic representation of the catalyzing effect of a L_a_ local arrangement of the membrane with 3 simple single-chain surfactants with different single chain lengths (Aa, Ba: small molecules can pass through the vesicle membrane; Ca: substance synthesized from Aa and Ba, trapped in the vesicle); R_a_, R_b_: possibly variable chemical radicals that do not interact with La.

This issue is addressed by the scenario of the emergence of a positive feedback process (following the constitution of a S_a_ site/C_a_ compound couple) in a lipidic vesicle-based model: as the bilayer membrane is composed of a mixture of various amphiphilic and hydrophobic compounds, there would have been the opportunity of a huge number of theoretically possible combinations in the arrangements of these compounds in the membrane. Among all these potential combinations, a specific L_a_ local arrangement of the inner surface of the membrane would have appeared, which is able to catalyze the combination of two substances A_a_ and B_a_ into a C_a_ compound (Figure 1).

**Figure 2:**
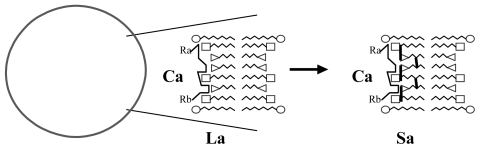
Schematic representation of the effect of Ca compound: stabilization of the La local arrangement into a stabilized Sa site.

In return, the Ca.compound would have been able to induce the transformation of the La local arrangement into a stabilized site Sa by the formation of covalent bonds (Figure 2).

**Figure 3:**
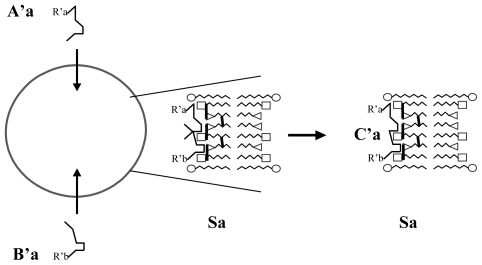
Schematic representation of the catalysis of a new molecule Ca by the same Sa site (Aa, Ba: small molecules can pass through the vesicle membrane; Ca: substance synthesized from Aa and Ba, trapped in the vesicle); R_a_, R_b_: possibly variable chemical radicals that do not interact with the Sa site.

S_a_/C_a_ couple is plausibly transmissible to the daughter vesicles because the self-replication of vesicles, as presently understood, is described as a process in which a growing vesicle transforms its shape from a sphere into a budded shape of two spheres connected by a narrow neck, and then splits into two spherical daughter vesicles [16]. Accordingly, both Ca and Sa are likely to be present in the daughter vesicles after division, particularly if Sa sites are randomly distributed on the inner surface of the membrane. This mechanism leads to the emergence of a strain of vesicles with a stable structure/function property. In addition, as new Sx/Cx couples can appear by chance, the emergence of numerous and diverse strains (e.g., more than one thousand with only 10 couples) allow Darwinian natural selection. Moreover, as each Sx site may catalyze not only one compound but a class of compounds (illustrated by the variable part of Cx: the Rx chemical radicals) the number of compounds per couple could be large (Figure 3).

The evolved information is represented by the S_x_/C_x_ couples in this model (Figure 2). This information is hereditary as it is plausibly transmissible to the daughter vesicles. Of course, in order to be useful to the survival of the vesicles, the C_x_ compounds should have had other specific properties in addition to their properties to induce the transformation of specific local arrangements L_x_ into sites S_x_: for example, while interfering with the vesicle membrane, they could increase the membrane stability, or the rate of division, or could allow the membrane adherence between two vesicles etc. The number of potential new strains depends on the number of S_x_/C_x_ couples and of their combinations. Thus, the potential of the model for the emergence of new distinct strains depends on the number of all possible combinations.

**Figure 4:**
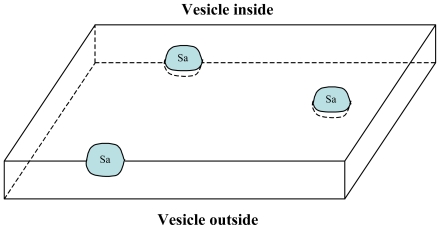
Schematic representation of the distribution of S_a_ sites on a local region of the inner surface of the vesicle membrane.

Actually, this latter number could even be increased by another very fruitful potential mechanism allowed by the model. Let us consider the inner surface of the membrane of a vesicle where a certain number of La local arrangements have been stabilized into S_a_ sites. The S_a_ sites are distributed over the inner surface of the vesicle (Figure 4).

**Figure 5:**
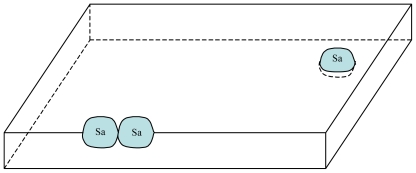
Schematic representation of the migration of S_a_ sites with the joining of two S_a_ sites.

However their respective localizations are not fixed: due to the fluidity of the membrane the sites are floating and can move on the inner surface. Thus two S_a_ sites can migrate and become very close together (Figures 5).

**Figure 6:**
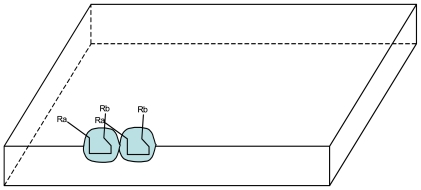
Schematic representation of the catalysis of a di-Ca compound by two adjacent Sa sites: a Ca compound binds on each of the two adjacent Sa sites.

**Figure 7:**
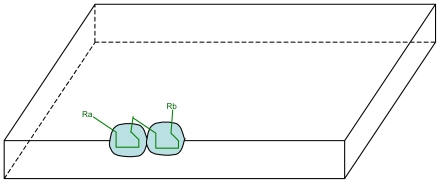
Schematic representation of the catalysis of a di-Ca compound by two adjacent Sa sites: synthesis of a di-Ca compound.

Two adjacent S_a_ sites could have catalyzed the synthesis of a di-C_a_ compound composed of two C_a_ compounds (Figure 6 and Figure 7).

**Figure 8:**
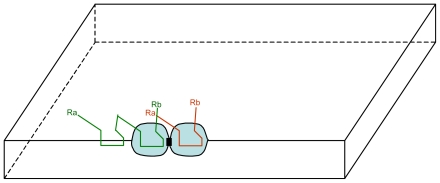
Schematic representation of the catalysis of the polymerization of Ca by a Sa-Sa site (here the synthesis of a tri-Ca): a di-Ca (in green) binds on the left Sa site of Sa-Sa while a Ca (in red) binds on the right Sa site.

**Figure 9:**
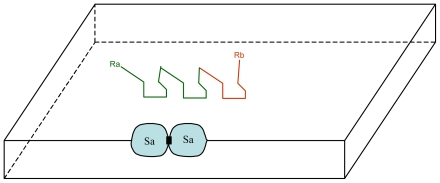
Schematic representation of the polymerization of Ca by a Sa-Sa site (here the synthesis of a tri-Ca): a synthesized tri-Ca is released by the Sa-Sa site.

Once synthesized di-C_a_ could have favoured the formation of covalent bonds between the two S_a_ sites, thus the formation of a S_a_-S_a_ site. When formed a S_a_-S_a_ site not only catalyzes the synthesis of di-C_a_ but can do more: the catalysis of the polymerization of C_a_ (Figure 8 and Figure 9).

However, steric effects could have occurred between the di-C_a_ and the membrane: the left C_a_ of di-C_a_ may have had space conflict with the membrane inner surface (Figure 8). Consequently, the probability that a di-C_a_ binds to the S_a_-S_a_ site as shown in Figure 7 would have been much higher than as shown in Figure 8.

Such example can be generalized to any S_x_ sites and their possible combinations. The combined S_x_-S_x_ sites would have been transmissible to the daughter vesicles according to the same process already described for the transmissibility of the S_x_/C_x_ couples (see above). This mechanism would have markedly increased the potential for the emergence of new distinct strains and would have led to the emergence of new promising properties.

### 3.2. Consequences of the Model: Homochirality and Polymers

The catalyzing effects of the sites would have crucial consequences by leading to enantioselectivity. Assuming that the first amino acids were synthesized through the catalyzing membrane sites and that the chiral centre carbon atom of the homochiral amino acids belonged to the catalysing domain, it would explain the emergence of the first L-amino acids. The asymmetric effect of homochiral dipeptide catalysis would have therefore yielded the stereospecific synthesis of D-tetroses [18]. The process could have explained the impressive and well-known homochirality of natural biopolymers. In addition, the possible polymerization of the amino acids according to the mechanism described above would have offered fruitful prospects.

### 3.3. Next Experiments

Experiments that prove the concept of a complex membrane catalyzing the synthesis of compounds that stabilize its local arrangements are yet to be developed. A first approach could be to study a large variety of vesicles with a bilayer membrane composed of a mixture of various amphiphilic and hydrophobic compounds [9]. Ideally, these latter compounds should be produced in conditions close to those of the hydrothermal vents such as the serpentinite-hosted Lost City Hydrothermal Field (see thereafter). Practically it would be possible to test whether these compounds could be synthesised in the conditions of continuous high-pressure flow reactors such as the one recently described and used by an American team [19]. If vesicles with bilayer complex membranes can be produced in these conditions, then the next steps would be to investigate the potential for the inner surface of such vesicle membranes to catalyze any kind of compounds from small molecules able to pass through the vesicle membrane and check whether the catalyzed compounds are trapped in the vesicle. Such a lipidic vesicle-based model with a complex membrane composed of mixtures of simple amphiphilic molecules is autotrophic as vesicles synthesize the compounds responsible for their specific properties. Others favor the same kind of lipidic vesicle-based model but an heterotrophic one as they hypothesize that the vesicle could have acquired complex nutrients such as nucleotides from the environment [20]. However, the problem of the abiotic synthesis of nutrients as complex as nucleotides remains unresolved.

## 4. The Constraints of Lipidic Vesicle-Based Models

### 4.1. Need for Lost City-Like Hydrothermal Vents

Lipidic vesicle models require specific conditions allowing the vesicles to emerge and persist long enough, including a continuous flow of matter and energy and the possibility for the waste products to be diluted in an open milieu so that the system is not hindered by their increasing concentration [9]. On today’s Earth there are places where such conditions are fulfilled: hydrothermal vents, particularly the serpentinite-hosted Lost City Hydrothermal Field [21]. The energy and the matter are provided by the vent fluids and the open milieu is the vast ocean. There are good arguments supporting the idea that many hydrothermal vents already existed on early Earth [22,23], some of which were deep-sea [21,24]. These deep-sea hydrothermal sites would have offered protective settings from the intensive solar UV radiations and the frequent massive meteorite impacts suffered by early Earth [22]. Compared to other studied sites, the Lost City Hydrothermal Field (LCHF) is an extreme endmember in which hydrothermal geochemistry is controlled primarily by serpentinization reactions that produce pH 9–11, <40–91 °C fluids enriched in H_2_ [21,25,26]. In addition, these fluids are enriched in calcium. Hence, mixing of these fluids with seawater results in the precipitation of large carbonate chimneys [27]. Actively venting chimneys and flanges are highly porous as they show fine anastomosing networks of carbonate lined with brucite, with tiny interconnected pores on the micrometer scale, indicating the mixing of seawater and hydrothermal fluids within the interior walls [21]. Fluids percolate through the carbonate anastomosing networks.

### 4.2. Synthesis of Lipid Compounds

It has already been recognized that vents systems were chemically reactive environments that constituted suitable conditions for sustained prebiotic syntheses [28]. At high temperatures, lipid compounds can be produced by aqueous Fischer-Tropsch-type (FTT) synthesis [29]. Such abiogenic production of short-chain hydrocarbons has been recently found at LCHF [25]. The millimolar concentrations of abiogenic CH_4_ present in the LCHF effluent could be at the origin of the carbon reduction in such hydrothermal systems [30]. Actually, fluids collected from the Rainbow and the Lost City hydrothermal fields were clearly enriched in organic compounds with a dominance of aliphatic hydrocarbons (C9-C14), aromatic compounds (C6-C16) and carboxylic acids (C8-C18) even though a mixed origin, *i.e.*, both biogenic and abiogenic, is probable [31]. Thermodynamic calculations demonstrate that biomass synthesis is most favourable at moderate temperatures such as at LCHF, where the energy contribution from HCO_3_ and H^+^ in seawater coupled to the reducing power in hydrothermal fluid are optimized [32]. Moreover experiments simulating molecular transport in elongated hydrothermal pore systems showed extreme accumulation of molecules in a wide variety of plugged pores [33]. New experiments demonstrated that thermal gradients across narrow channels can provide the energy necessary to concentrate dilute molecular solutions and thus allow the self-assembly of lipidic vesicles from an initially dilute solution [34]. Vesicles with membranes composed of bi-layers from mixtures of amphiphilic and hydrophobic molecules could have formed from the organic compounds present locally at high concentrations. The stability of bilayer lipidic membranes at high pressure and temperature is nevertheless still debated. Experiments have shown that bilayers formed of simple amphiphiles are extremely fragile: high pH, ionic strength, high temperatures (even 45 °C) will destroy them [35,36]. However primitive membranes would have been composed of a diverse mixture of amphiphiles. This mixt character may have imparted essential stability to primitive membranes [36,37]. Furthermore, polycyclic aromatic hydrocarbon (PAH) may have contributed to stabilizing them as cholesterol stabilizes cell membranes of extant organisms today [35,31]. Recent experiments show that monoglycerides are synthesized under hydrothermal conditions by simple condensation reactions which represent a plausible step in the self-assembly of protocellular structures toward boundary membranes that would be stable over a range of pH values in the salty seas of the prebiotic environment. Actually, in these experiments, no salts were present either during the synthesis or during the formation of bilayers, which occurs at pH 8.5. The authors justified their conclusions by the fact that monoglycerides are virtually immune to the effect of pH and divalent cations, because they do not have ionic head groups that can interact with cations in solution [38]. Finally, this membrane stability problem would have been a strong selection factor among all the possible sorts of vesicles with heterogeneous membranes.

### 4.3. The Phosphorus Issue

The hypothesis that lipidic vesicles were produced on early Earth 4,000 million years ago at sites such as LCHF makes deep-sea hydrothermal sites the best candidates to be at the origin of evolution and sets new constraints. Heterotrophic scenarios that call for abiotic synthesis of nucleotides at hydrothermal sites require a source of phosphorus, whereas mid-ocean ridges are not a source, but a sink of phosphorus [39]. This is a major constraint, and scenarios based on acetyl phosphate raise the same issue [40]. The answer may be that, at that remote time, arsenic was used instead of phosphorus, as claimed recently. However the interpretation by the authors of the data from the GFAJ-1 bacterial strain, isolated from Mono Lake, California [41] is very much debated [42,43] and abiotic synthesis of nutrients as complex as nucleotides remains an unresolved question, be it phosphorus or arsenic-based.

### 4.4. Need for a Tectonic Plate Dynamic Regime?

Celestial bodies considered as candidates for the emergence of evolution should plausibly have a very long-standing process of plate tectonics in order to allow the occurrence and maintenance of deep-sea hydrothermal sites such as LCHF. These constraints would add to classical requirements, e.g., basic ingredients such as reduced carbon-based molecules and liquid H_2_O [44]. The requirement of the existence of plate tectonics would suggest Mars as a possible cradle of evolution. Mars is very likely to be in a stagnant-lid regime now, and has been for much of its history. However, if surface water was present in its early history, it may have been in the active-lid regime at that time [45,46]. In addition, lines of evidence suggest that hydrothermal conditions might have existed on Mars: although an igneous origin cannot be excluded, the formation of the carbonate-rich outcrops recently identified by the Spirit Rover could be due to aqueous processes under hydrothermal conditions in the planets first billion years [47]. Moons like Europa could be questioned too [45]. However the existence of hydrothermal vents is conceivable on Europa [48]: in particular tidal flexing on Europa may generate heat of the same order as present-day radiogenic heat flux at the Earth’s surface that may drive hydrothermal fluid flows [49].

### 4.5. Origin of Evolution *versus* Origin of Life: Consequences

Finally, within such a shift of paradigm in the approach of the primordial ancestor, there would be further consequences. In particular the search for biosignatures would be affected, not when biosignatures are supposed to be related to relatively high-evolving microorganisms, but in the search for the primordial ancestor. Even with theories that pre-supposed the abiotic synthesis of biological polymers in the early Earth environment, such as the “prevolutionary dynamics” theory [50], the usual distinctive features between living systems and non living ones could not be considered any more: the distinctive features would now be between systems with evolvable capacity and systems without. In the presented lipidic vesicle-based model, the only differences are the membrane sites and their ligands but of course they are not yet identified. However, the model implies the enanthioselectivity of the synthesized compounds which represents a specific signature. The paradigm shift in the approach to the question of the primordial ancestor could have a negative consequence: the public may better accept the funding of research on the “origin of life” than on the “origin of evolution”, particularly in the US where there are religious groups and lobbies very reluctant to accept the theory of evolution. This may raise issues concerning US research and NASA programmes.

## 5. Conclusions

There is no point in attempting to define life because of the irreducible metaphysical aspect of the concept. Instead it seems more appropriate to focus on the process of evolution, as the source of the primordial ancestor on Earth and presumably similar systems elsewhere. The consensus to be reached in the quest for the primordial ancestor should be in explaining the minimal processes that allowed evolution to emerge and persist. A process is proposed here that requires only three conditions without necessarily involving biopolymers.
